# Potential of Alkalization Therapy for the Management of Metastatic Pancreatic Cancer: A Retrospective Study

**DOI:** 10.3390/cancers16010061

**Published:** 2023-12-21

**Authors:** Masahide Isowa, Reo Hamaguchi, Ryoko Narui, Hiromasa Morikawa, Toshihiro Okamoto, Hiromi Wada

**Affiliations:** 1 Japanese Society on Inflammation and Metabolism in Cancer, 119 Nishioshikouji-cho, Nakagyo-ku, Kyoto 604-0842, Japan; misowa@scim.or.jp (M.I.); rnarui@scim.or.jp (R.N.); hmorikawa@scim.or.jp (H.M.); wadah@kuhp.kyoto-u.ac.jp (H.W.); 2 Department of Thoracic and Cardiovascular Surgery, Cleveland Clinic, Cleveland, OH 44195, USA; okamott@ccf.org; 3 Department of Inflammation and Immunology, Lerner Research Institute, Cleveland Clinic, Cleveland, OH 44195, USA; 4 Transplant Center, Cleveland Clinic, Cleveland, OH 44195, USA

**Keywords:** pancreatic cancer, cancer metabolism, alkalization therapy, tumor microenvironment, urine pH

## Abstract

**Simple Summary:**

In recent years, the acidic tumor microenvironment has been receiving increased attention as a target for cancer treatment. In our previous retrospective studies, we observed that alkalization therapy significantly increased urine pH in patients, which correlated with favorable cancer treatment outcomes. However, the effectiveness of alkalization therapy in conjunction with chemotherapy for pancreatic cancer patients remained unclear, as did the correlation between increased urine pH and extended survival. Therefore, we conducted this retrospective study to investigate the effects of integrating alkalization therapy with current treatments for pancreatic cancer patients, focusing on whether the synergetic effects of pH regulation through alkalization therapy enhance the effectiveness of cancer treatment. In this study, we noted a trend in which longer patient survival was associated with a higher average urine pH, suggesting a correlation between the effectiveness of alkalization therapy and increased urine pH levels.

**Abstract:**

Current treatments for patients with pancreatic cancer offer limited benefits. In this study, we applied alkalization therapy, which was efficacious for other solid tumors at our clinic, to stage 4 pancreatic cancer patients, and investigated its effect on disease prognosis. Patients with metastatic pancreatic cancer who were treated at Karasuma Wada Clinic in Kyoto, Japan, between January 2011 and April 2022, were included in the study. All patients received alkalization therapy (a combination of an alkaline diet, bicarbonate, and citric acid administration), alongside standard chemotherapy. Urine samples were collected to assess urine pH as a marker of whole-body alkalization. In the 98 patients analyzed, the median overall survival (OS) from the time of diagnosis was 13.2 months. Patients with a mean urine pH of 7.5 or greater had a median OS of 29.9 months, compared with 15.2 months for those with a mean urine pH of 6.5 to 7.5, and 8.0 months for those with a mean urine pH of less than 6.5, which suggests a trend of a longer OS in patients with a higher urine pH (*p* = 0.0639). Alkalization therapy may offer a viable approach to extending the survival of stage 4 pancreatic cancer patients, who typically have an unfavorable prognosis.

## 1. Introduction

Pancreatic cancer is a devastating disease with a rapidly increasing incidence and a very high mortality rate [1]. In Japan, the incidence rate is 34.8 per 100,000 people, and the 5-year relative survival rate is 8.5% [2]. Moreover, it is the fourth leading cause of cancer death in Japan [2]. Most patients with pancreatic cancer have local invasion and distant metastasis at the time of diagnosis, making curative resection impossible in many cases [3]. Even if surgery is performed, the recurrence rate is high, and the prognosis is unfavorable [4]. To overcome this situation, either the discovery of a clinically applicable method for detecting tumors at a resectable stage or the establishment of an effective nonsurgical treatment to prevent tumor progression is needed, and, to date, various studies have been conducted on these points [5,6]. Among the currently available treatments, nonsurgical treatments, such as chemotherapy and radiotherapy, have continued to provide some extension of survival time for pancreatic cancer patients, but the results are still not satisfactory [7].

We have been focusing on the tumor microenvironment (TME) surrounding malignant tumors as a possible therapeutic target. Our reasons are as follows: (1) malignant tumors use glycolysis for their cellular metabolism and release more protons into the extracellular space than normal cells, lowering the pH of the TME to acidic levels [8]; and (2) the acidic TME attracts inflammatory cells that cause chronic inflammation and create a more favorable environment for tumor growth [9]. Based on these molecular and cell biological findings, we hypothesized that preventing malignant tumors from creating an acidic TME and reversing it by raising the pH of the TME would inhibit tumor growth and decrease the tumor’s resistance to anticancer drugs [10]. We then devised an original treatment method that we named alkalization therapy, which can be applied clinically based on this idea. Alkalization therapy is a simple therapy performed by shifting the patient’s diet to one that is expected to alkalize the whole body, and, furthermore, its effect is enhanced by combining it with the administration of alkalizing agents, such as bicarbonate and citric acid [11,12,13]. We have obtained favorable results using alkalization therapy at our clinic in patients with various types of cancers, such as malignant lymphoma, gastric cancer, small-cell lung cancer, and breast cancer [14]. In addition, we previously conducted a study on patients with advanced or recurrent pancreatic cancer at our clinic and reported that the combination of chemotherapy and alkalization therapy showed a significant advantage over chemotherapy alone, with a median overall survival (OS) of 15.4 months (the combination treatment group) vs. 10.8 months (the chemotherapy alone group) (*p* < 0.005) [15].

However, one problem is the method of evaluating whether or not alkalization therapy is actually contributing to increasing the pH of the TME. We previously reported the association between urine pH and antitumor effects in our evaluation of the potential use of urine pH as an indicator of whether or not alkalization therapy is being performed adequately. In a study on patients with hepatocellular carcinoma, in which alkalization therapy was monitored using urine pH, we found that the median OS from the initiation of treatment was not reached for patients who achieved a urine pH of at least 7.0 (n = 12; 95% confidence interval (CI) = 3.0—not reached), indicating their significantly longer survival than patients who had a lower urine pH (15.4 months; n = 17; 95% CI = 5.8—not reached, *p* < 0.05) [16].

Similar to a previous study that included patients with metastatic and recurrent pancreatic adenocarcinoma, we focused on a cohort of patients with stage 4 metastatic pancreatic adenocarcinoma, and re-examined the efficacy of alkalization therapy, a nonsurgical treatment that has shown favorable outcomes at our clinic for various solid tumors. Furthermore, we investigated the effect of urine pH levels on prognosis improvement by grouping the patients into three categories based on their urine pH values.

## 2. Patients and Methods

### 2.1. Study Design

**Figure 1:**
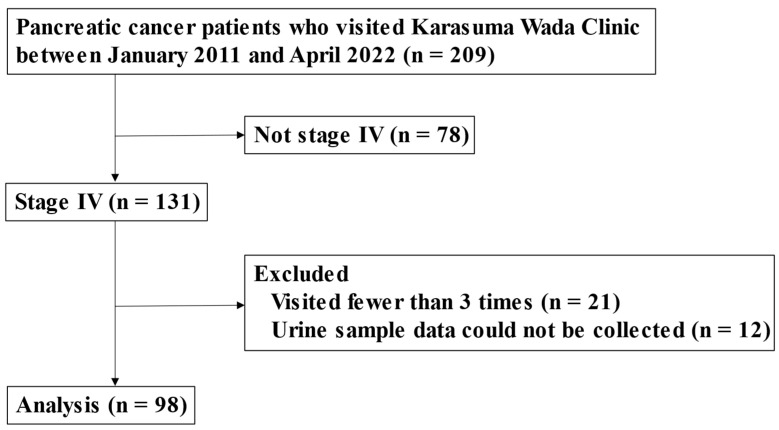
Flowchart of this study.

This study was a retrospective analysis performed to assess the potential effects of alkalization therapy on patients with stage 4 metastatic pancreatic adenocarcinoma. The patients were treated at Karasuma Wada Clinic in Kyoto, Japan, between 1 January 2011, and 30 April 2022. Data were collected from the clinic’s medical records. All participants received alkalization therapy, which consisted of an alkalizing diet and the oral administration of alkalizing agents (bicarbonate and citric acid). The participants were outpatients who were able to follow dietary guidance. Alkalization therapy was performed in conjunction with standard chemotherapies for pancreatic cancer. A flowchart of patient inclusion/exclusion is shown in Figure 1.

Above is a flowchart showing the number of patients analyzed in this study. Of the 209 patients with pancreatic adenocarcinoma who visited Karasuma Wada Clinic between January 2011 and April 2022, 131 were diagnosed as having stage 4 cancer. After excluding 21 patients who visited fewer than 3 times and 12 patients for whom urine samples could not be collected, a total of 98 patients were included in the analysis. The study population included 25 patients from our previous study [15].

### 2.2. Alkalization Therapy

Alkalization therapy was defined as a combination of an alkaline diet and the oral administration of bicarbonate (3.0–5.0 g/day) and/or citric acid (3.0–6.0 g/day). The alkaline diet consisted of a large amount of fruits and vegetables and minimal amounts of meat and dairy products. Patients were instructed to consume at least 400 g of fruits and vegetables daily, while avoiding meat and dairy products. Patients documented their meals for the initial 4 weeks of the diet. At each visit, a doctor or nurse reviewed the patient’s meal logs to ensure dietary adherence and offered advice based on the meal logs. Ultimately, the final dietary choices were at the discretion of the patients.

### 2.3. Assessment Procedures

Data were analyzed on 30 September 2023. The median OS from the time of the initial diagnosis was calculated. Urine samples were collected during regular patient visits, which occurred at a minimum of once every 2 months and up to twice a month. The OS from the time of initial diagnosis was then compared among the following 3 groups: Group 1 patients with a mean urine pH of 7.5 or higher, Group 2 patients with a mean urine pH of 6.5 or higher but less than 7.5, and Group 3 patients with a mean urine pH of less than 6.5.

### 2.4. Statistical Analyses

For each patient, mean urine pH values were measured for all collected urine samples, from the patient’s initial visit to Karasuma Wada Clinic through to 30 April 2022. Mean values and their standard deviations were calculated for each group in the dataset. Differences between groups were assessed using 2-sided *p*-values, and a *p*-value of less than 0.05 was considered to indicate a statistically significant difference between groups. Statistical analyses were conducted using Easy R (version 1.61; Saitama Medical Center, Jichi Medical University, Saitama, Japan), a graphical user interface based on R (version 4.22; The R Foundation for Statistical Computing, Vienna, Austria) [17].

## 3. Results

### 3.1. Patient Characteristics

Between 1 January 2011 and 30 April 2022, 209 patients with pancreatic cancer visited Karasuma Wada Clinic. Of these, 131 patients were diagnosed as having stage 4 cancer. After excluding 21 patients who visited fewer than three times and 12 patients for whom urine samples could not be collected, a total of 98 patients were included in the analysis (Figure 1). The cohort consisted of 53 men and 45 women. The mean age at their first visit to Karasuma Wada Clinic was 64.5 years, with a range of 27 to 88 years. All of the patients were outpatients (performance status (PS): 0–1) and underwent alkalization therapy, as shown in Table 1. The median age of the assessed patients was in the 60s for all three groups, and there were slightly more men in all groups. Few patients in Groups 2 and 3 had a prior history of malignancy and had undergone radiotherapy for pancreatic cancer. The PS was slightly less favorable in Group 1 than in the other groups. First-line chemotherapy regimens were mostly gemcitabine hydrochloride + nab-paclitaxel combination therapy (GEM + nabPTX) or folinic acid, fluorouracil, and irinotecan oxaliplatin therapy (FOLFIRINOX) in Group 1, whereas they were more diverse in Groups 2 and 3. Moreover, there were more patients who did not receive any chemotherapy in Group 3.

### 3.2. OS of Patients with Different Urine pHs

**Figure 2:**
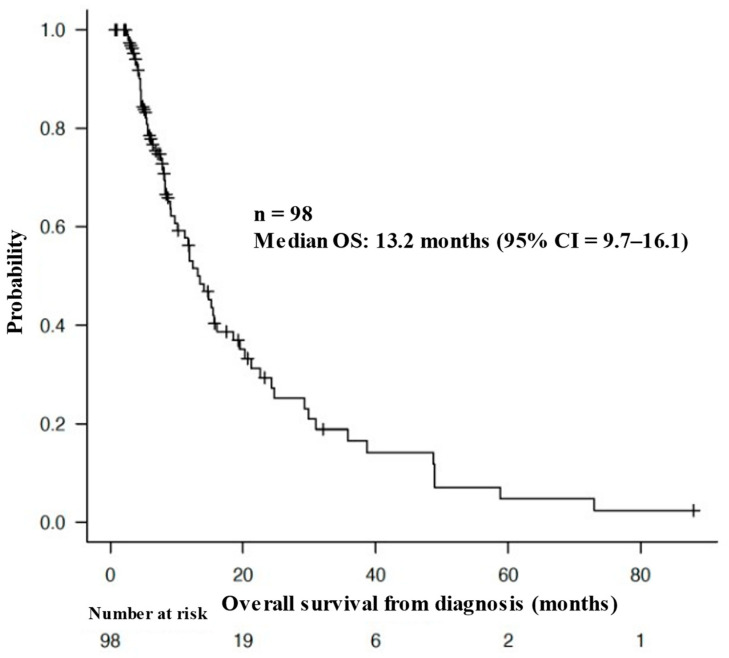
Overall survival of the patients from the time of diagnosis.

**Figure 3:**
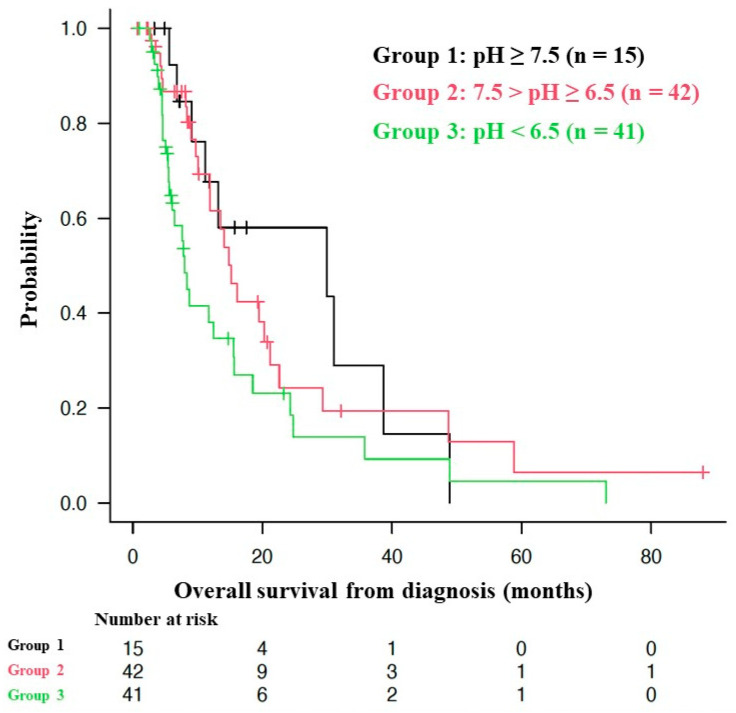
Association between overall survival and urine pH.

The median OS of all patients from the time of diagnosis was 13.2 months (95% CI = 9.7–16.1 months), as shown in Figure 2. Figure 3 shows the Kaplan–Meier curves for the OS of patients stratified by mean urine pH levels, as follows: those with a mean urine pH of 7.5 or higher, those with a mean urine pH of 6.5 to less than 7.5, and those with a mean urine pH of less than 6.5. The median OS was 29.9 months for the 15 patients with a mean urine pH of 7.5 or higher (95% CI = 9.1–38.7), 15.2 months for the 42 patients with a pH between 6.5 and less than 7.5 (95% CI = 10.1–21.2), and 8.0 months for the 41 patients with a pH of less than 6.5 (95% CI = 5.6–15.5). Although these results did not reach statistical significance, there was a discernible trend, i.e., higher urine pH correlated with extended OS (*p* = 0.0639).

Above is a Kaplan–Meier curve of the overall survival (OS) of the patients from the time of diagnosis. The median OS of all patients from the time of diagnosis was 13.2 months (95% CI = 9.7–16.1 months).

Figure 3 shows Kaplan–Meier curves of the overall survival (OS) from the start of alkalization therapy of patients with a mean urine pH of ≥ 7.5 (Group 1), 7.5 > pH ≥ 6.5 (Group 2), and pH < 6.5 (Group 3). For the pH ≥ 7.5 group (Group 1, n = 15), the median OS was 29.9 months (95% confidence interval: CI = 9.1–38.7); for the 7.5 > pH ≥ 6.5 group (Group 2, n = 42), the median OS was 15.2 months (95% CI = 10.1–21.2); and for the pH < 6.5 group (Group 3, n = 41), the median OS was 8.0 months (95% CI = 5.6–15.5). Of the 63 deaths, only 1 was owing to head trauma after a fall. The other 62 deaths were owing to the primary disease. 

## 4. Discussion

The increase in the number of pancreatic cancer patients is a global trend, and the incidence of pancreatic cancer is expected to increase further in the future. The reason for this is that the risk of pancreatic cancer increases with age, and the proportion of the population aged 65 years and older is expected to double globally in the next few decades. Therefore, the incidence of pancreatic cancer is expected to continue to increase for the next several decades [1]. Moreover, pancreatic cancer tends to have an unfavorable prognosis because (1) most patients have advanced cancer at the time of diagnosis, (2) early vascular and neural invasion and distant metastasis are common, (3) the effects of anticancer chemotherapy and radiotherapy are limited, (4) there is a complex TME (with various interactions between neoplastic and stromal cells within the TME), and (5) multiple genetic and acquired mutations can cause the disease, i.e., there are few prevalent genetic mutations, and none of the most commonly mutated genes are currently druggable [18,19]. Particularly for patients with stage 4 pancreatic cancer with distant metastasis, current treatments, such as systemic chemotherapy and radiotherapy, have reached their limits in terms of prolonging survival, and more innovative and effective treatments are needed.

At our clinic, we encounter many patients with advanced-stage cancers that are not suitable for curative surgery, systemic chemotherapy, or radiotherapy. Therefore, we perform alkalization therapy, which is based on the concept of increasing the pH of the whole body and the TME to achieve antitumor effects, and have achieved favorable results [11,14,15]. Whether alkalization therapy has been performed adequately or not is assessed by measuring the patient’s urine pH, and we have previously reported that higher urine pH is associated with longer survival in hepatocellular carcinoma patients and pancreatic cancer patients [15,16]. In the present study, we designed a single-center, retrospective, observational study to investigate whether alkalization therapy can prolong the survival of stage 4 pancreatic cancer patients. We furthermore investigated the association between urine pH levels and the prognosis of the patients.

Involvement of the TME in tumor growth and proliferation has long been recognized [20]. The fact that cancer cells depend on glycolysis for their energy metabolism is a concept that was initially proposed by Otto Warburg et al. (the Warburg effect) [21]. As a result, protons are released from tumor cells into the extracellular space, making the TME acidic [22]. Acidification of the TME leads to the attraction and infiltration of inflammatory cells, which further exacerbate chronic inflammation [23]. This promotes angiogenesis and blood flow imbalance in the tumor, resulting in chronic hypoxia and a vicious cycle of the activation of glycolysis, TME acidification, chronic inflammation, and then the further activation of glycolysis [21]. Previous basic science studies have shown that the pH around normal cells surrounding a tumor is 7.2 to 7.4, whereas that around tumor cells is 6.6 to 7.0 [24,25,26,27]. The aim of alkalization therapy is to break or reverse this vicious cycle of glycolysis, TME acidification, chronic inflammation, and further activation of glycolysis, and to achieve antitumor effects by increasing the pH of the TME [28,29]. As in our previous reports, this paper proposes an important paradigm shift, i.e., that not only the malignant tumor itself but also the TME should be considered as a major therapeutic target. A previous study focused on the TME in mouse models of end-stage pancreatic cancer, which showed that oral administration of sodium potassium citrate increased HCO_3_^−^ concentration in blood, HCO_3_^−^ concentration and pH in urine, and neutralized the tumor extracellular pH [13]. In addition, we should consider the other ideas of alkalization therapy that have been proposed to date. The use of proton pump inhibitors (PPIs) to alkalize the TME has been attracting attention. It has been observed that PPIs prevent tumor cells from acquiring resistance to cytotoxic anticancer drugs and induce the apoptosis of tumors in animal models and cultured cells of malignant melanoma, adenocarcinoma, and malignant lymphoma [30,31,32]. Furthermore, in a large retrospective observational study that investigated the association between PPI exposure and breast cancer incidence, patients diagnosed with breast cancer had 25% less exposure to PPIs than the matched control group (95% CI = 0.72–0.78) [33], and in a prospective cohort study that compared the incidence of breast cancer between PPI users and non-users, the adjusted hazard ratio for PPI users was 0.32 (95% CI = 0.20–0.49) [34], with both studies concluding that PPI intake may reduce the risk of breast cancer. In a randomized controlled trial study using PPIs as an alkalizing agent, PPI was combined with chemotherapy in patients with metastatic breast cancer, and antitumor effects were observed [35]. In addition to PPIs, in a study using water with added alkalizing agent, it was found that the progression of prostate cancer in the transgenic adenocarcinoma of the mouse prostate mouse model was suppressed by drinking alkaline water [36]. Similarly, there is also a report that alkaline water significantly inhibited tumor growth in a mouse model of malignant melanoma [37].

All 98 patients who visited our clinic more than three times and had their urine pH measured had stage 4 pancreatic cancer with distant metastasis. All of them were instructed on how to follow an alkaline diet (concept sharing and nutritional guidance), but the final dietary preferences and choices were left to the patients. We judged whether alkalization therapy was being performed according to the instructions or not by measuring the patient’s urine pH. Theoretically, a higher urine pH indicates successful implementation of alkalization therapy. In this study, only 22 patients achieved an average urine pH of 7.5 or higher. This shows that the degree of alkalization achieved varies from patient to patient, even though all of them were instructed on how to perform alkalization therapy. This also shows that simply sharing the concept of and providing guidance on alkalization therapy does not necessarily result in alkalization of the whole body.

The first-line chemotherapy recommended by the Guidelines for Pancreatic Cancer with Distant Metastasis in Japan is either FOLFIRINOX or GEM + nabPTX [7]. A basis for this recommendation is a single-arm phase two trial of modified FOLFIRINOX therapy, which enrolled 69 patients and reported a median OS of 11.2 months (95% CI = 9.0—not reached) [38]. However, the regimen was associated with serious adverse events and death owing to treatment, and the authors cautioned readers about the toxicity of this regimen in their paper [38]. Another basis is a single-arm phase 2 trial of gemcitabine hydrochloride + nab-paclitaxel combination therapy, which enrolled 34 patients and reported a median OS of 13.5 months (95% CI = 10.6—not reached). This regimen was reported to have a lower incidence of adverse events than FOLFIRINOX therapy [39].

In the present study, the median OS from the time of diagnosis of the patients who received alkalization therapy in addition to the standard treatment was 13.2 months (95% CI = 9.7–16.1), which was comparable or superior to that of patients who received the two regimens recommended by the Guidelines for Pancreatic Cancer with Distant Metastasis in Japan (FOLFIRINOX or gemcitabine hydrochloride + nab-paclitaxel combination) for the treatment of pancreatic cancer. Moreover, the median OS of the group with an average urine pH of 7.5 or higher was 29.9 months (95% CI = 9.1–38.7), which can be considered as an outstanding result exceeding the prognosis of patients receiving the standard treatment. By comparing the median OS of the three groups divided by average urine pH levels, i.e., a pH of 7.5 or higher, 6.5 or higher but less than 7.5, and less than 6.5, we observed that longer patient survival depended on a higher average urine pH, which suggested that, despite the absence of a statistical difference (*p* = 0.0639) [40], there was a correlation between average urine pH and the achievement of the objective of alkalization therapy. This remarkable result suggests that, in the treatment of malignant tumors, although targeting the behavior and genetic mutations of tumor cells is certainly important, alkalizing and modifying the TME can amplify the antitumor effects of standard treatments. Furthermore, the TME can also act as a sufficient therapeutic target itself, and hence alkalization therapy is a promising treatment method that is gentle on the patient’s body, does not cost much, and is expected to lead to a paradigm shift.

This study has several limitations. First, it was a single-center retrospective observational study, and we could not compare the outcomes with a non-intervention group of patients who did not receive alkalization therapy. This is because all patients in this study were introduced to alkalization therapy (concept sharing and nutritional guidance) at our clinic and underwent alkalization therapy, so there was no control group that did not receive alkalization therapy. In addition, to further confirm the efficacy of alkalization therapy, a standardized protocol for alkalization therapy should be created, and a prospective cohort study should be conducted at multiple centers in the future. Second, there was a large variation in patient backgrounds, mainly in the time from diagnosis to the start of alkalization therapy. Although this study was limited to patients with stage 4 pancreatic cancer with distant metastasis, there may be a need to adjust for background factors, such as sex, location of metastasis, types of chemotherapy regimens, and other concurrent treatments. Furthermore, it would be ideal if alkalization therapy could be started immediately after disease diagnosis, as this may demonstrate the effect of alkalization therapy more clearly. Finally, we used urine pH as a target marker for alkalization therapy. The pH of body fluids, such as urine and blood, changes over time owing to various factors, and may vary greatly depending on underlying diseases or medications. In addition, the degree of pH change of the TME may vary depending on the malignancy and number of tumor cells. In the future, we hope to identify all of the factors affecting the pH of the TME, clarify their interactions, and draw conclusions based on them.

## 5. Conclusions

We demonstrated the potential of alkalization therapy in combination with standard treatments to prolong the survival of patients with stage 4 pancreatic cancer with distant metastasis, which has a very unfavorable outcome. We also found that an increase in urine pH may contribute to further survival extension in patients with advanced pancreatic cancer. Further studies are required to confirm the clinical efficacy of alkalization therapy in preventing TME acidification and achieving antitumor effects.

## Figures and Tables

**Table 1:** Patient characteristics.

	Group 1 (n = 15) Urine pH ≥ 7.5			Group 2 (n = 42) 7.5 ≥ Urine pH > 6.5			Group 3 (n = 41) 6.5 ≥ Urine pH		
Age (years)	65.3 (45–85)			62.6 (33–85)			66.1 (27–88)		
Sex (%)									
Men	9 (60.0)			23 (54.8)			21 (51.2)		
Women	6 (40.0)			19 (45.2)			20 (48.8)		
Performance status (%)									
0	8 (53.3)			30 (71.4)			25 (61.0)		
1	7 (46.7)			12 (28.6)			16 (39.0)		
Comorbidities (%)									
Hypertension	3 (20.0)			7 (16.7)			8 (19.5)		
Hyperlipidemia	3 (20.0)			8 (19.0)			4 (9.8)		
Diabetes	2 (13.3)			10 (23.8)			5 (12.2)		
Hyperuricemia	1 (6.7)			2 (4.8)			3 (7.3)		
Arrhythmia	2 (13.3)			2 (4.8)			1 (2.4)		
Angina	0			2 (4.8)			0		
COPD	0			2 (4.8)			0		
Depression	2 (13.3)			0			1 (2.4)		
Others	3 * (20.0)			5 ** (11.9)			6 *** (14.6)		
Malignant disease (past history)	0			5 (11.9)			5 (12.2)		
None	5 (33.3)			16 (38.1)			20 (48.8)		
Radiation (%)									
Yes	0 (0.0)			2 (4.8)			5 (12.2)		
No	15 (100.0)			40 (95.2)			36 (87.8)		
Chemotherapy (%)	1st line	2nd line	3rd line	1st line	2nd line	3rd line	1st line	2nd line	3rd line
FOLFIRINOX	4 (26.7)	2 (13.3)	0	4 (9.5)	2 (4.8)	0	4 (9.8)	1 (2.4)	0
FOLFIRI	0	2 (13.3)	0	0	0	0	0	0	0
Gemcitabine plus nab-paclitaxel	8 (53.3)	0	0	14 (33.3)	5 (11.9)	1 (2.4)	12 (29.3)	2 (4.9)	0
Gemcitabine plus erlotinib	0	0	0	3 (7.1)	1 (2.4)	0	2 (4.9)	2 (4.9)	1 (2.4)
Gemcitabine plus S-1	0	0	0	4 (9.5)	0	0	4 (9.8)	1 (2.4)	0
Gemcitabine	2 (13.3)	1 (6.7)	0	8 (19.0)	3 (7.1)	0	5 (12.2)	1 (2.4)	0
S-1	0	2 (13.3)	1 (6.7)	3 (7.1)	4 (9.5)	0	2 (4.9)	6 (14.6)	0
Erlotinib	0	0	0	4 (9.5)	0	0	3 (7.3)	0	1 (2.4)
Unknown	0	0	1 (6.7)	0	2 (4.8)	0	2 (4.9)	0	0
None	1 (6.7)	8 (53.3)	13 (86.7)	2 (4.8)	25 (59.5)	41 (97.6)	7 (17.1)	28 (68.3)	39 (95.1)

* Asthma, dementia, and rheumatoid arthritis; ** cerebral infarction, dementia, hepatitis B virus, hepatitis C virus, and interstitial pneumonia; *** asthma, cerebral infarction, valvular heart disease, hepatitis B virus, hyperthyroidism, and deep vein thrombosis. COPD, chronic obstructive pulmonary disease; FOLFIRINOX, leucovorin, fluorouracil, irinotecan, and oxaliplatin; FOLFIRI, leucovorin, fluorouracil, and irinotecan.

## Data Availability

Data are contained within the article.
